# Combating Traumatic Brain Injury: A Dual-Mechanism Hydrogel Delivering Salvianolic Acid A and Hydroxysafflor Yellow A to Block TLR4/NF-κB and Boost Angiogenesis

**DOI:** 10.3390/polym17141900

**Published:** 2025-07-09

**Authors:** Guoying Zhou, Yujia Yan, Linh Nguyen, Jiangkai Fan, Xiao Zhang, Li Gan, Tingzi Yan, Haitong Wan

**Affiliations:** 1 College of Life Science, Zhejiang Chinese Medical University, Hangzhou 310053, China; gyzhou87@126.com (G.Z.); yanyujiay@163.com (Y.Y.); 19818503486@163.com (J.F.); 19883206056@163.com (X.Z.); hz0113@126.com (L.G.); 2 Division of Biomaterials and Tissue Engineering, Eastman Dental Institute, University College London, London WC1E 6BT, UK; l.nguyen@ucl.ac.uk; 3 Royal Free Hospital, Pond Street, London NW3 2QG, UK; 4 College of Material, Chemistry and Chemical Engineering, Hangzhou Normal University, Hangzhou 311121, China; 5 Institute of Cardio-Cerebrovascular Disease, Zhejiang Chinese Medical University, Hangzhou 310053, China

**Keywords:** traumatic brain injury, hydrogel, anti-inflammatory, angiogenesis, salvianolic acid A, hydroxysafflor yellow A

## Abstract

Traumatic brain injury (TBI) leads to severe neurological dysfunction, disability, and even death. Surgical intervention and neurorehabilitation represent the current clinical management methods, yet there remains no effective treatment for recovery after TBI. Post-traumatic hyperinflammation and vascular injury are the key therapeutic challenges. Therefore, a novel-designed multifunctional HT/SAA/HSYA hydrogel based on hyaluronic acid (HA) co-loaded with salvianolic acid A (SAA) and hydroxysafflor yellow A (HSYA) was developed in order to simultaneously target inflammation and vascular injury, addressing key pathological processes in TBI. The HT hydrogel was formed through covalent cross-linking of tyramine-modified HA catalyzed by horseradish peroxidase (HRP). Results demonstrated that the HT hydrogel possesses a porous structure, sustained release capabilities of loaded drugs, suitable biodegradability, and excellent biocompatibility both in vitro and in vivo. WB, immunofluorescence staining, and PCR results revealed that SAA and HSYA significantly reduced the expression level of pro-inflammatory cytokines (IL-1β and TNF-α) and inhibited M1 macrophage polarization through the suppression of the TLR4/NF-κB inflammatory pathway. In vivo experiments confirmed that the HT/SAA/HSYA hydrogel exhibited remarkable pro-angiogenic effects, as evidenced by increased expression of CD31 and α-SMA. Finally, H&E staining showed that the HT/SAA/HSYA hydrogel effectively reduced the lesion volume in a mouse TBI model, and demonstrated more pronounced effects in promoting brain repair at the injury site, compared to the control and single-drug-loaded hydrogel groups. In conclusion, the HT hydrogel co-loaded with SAA and HSYA demonstrates excellent anti-inflammatory and pro-angiogenic effects, offering a promising therapeutic approach for brain repair following TBI.

## 1. Introduction

Traumatic brain injury (TBI) represents a significant neurological condition caused by external mechanical forces, including impact, vibration, or penetration of the brain. It is a leading cause of disability and death worldwide [1]. It leads to the disruption of the blood–brain barrier, loss of neural tissue, inflammation, and vascular damage, ultimately contributing to long-term neurological disorders [2,3,4]. Vascular injuries can lead to an insufficient supply of oxygen and nutrients [5,6]. Therefore, reducing neuroinflammation and promoting angiogenesis after TBI are crucial for minimizing damage and promoting repair [6,7].

Recently, traditional Chinese medicine (TCM) has garnered increasing attention for its demonstrated efficacy in treating various diseases [8]. Notably, Salvia miltiorrhiza and Safflower, a classic herbal pair, are widely employed in managing cardiovascular and neurological disorders owing to their synergistic effects in promoting blood circulation and reducing inflammation [9,10]. Salvianolic acid A (SAA), a water-soluble bioactive compound isolated from Salvia miltiorrhiza, possesses multifaceted pharmacological properties, including anti-inflammatory, antioxidant, and anti-fibrotic activities [11,12,13]. Zhang et al. demonstrated that SAA alleviates cerebral ischemia–reperfusion injury by inhibiting NF-κB p65 activation and suppressing the expression of matrix metalloproteinase-9 (MMP-9) in the rat brain, thereby attenuating the inflammatory response, reducing infarct volume, and improving neurological deficit scores, ultimately mitigating cerebral ischemia–reperfusion injury [14]. Ling et al. reported that SAA suppressed microglia-mediated neuroinflammation by selectively inhibiting the MyD88-dependent TLR2/4 signaling pathway in an acute cerebral ischemia–reperfusion model [15]. On the other hand, hydroxysafflor yellow A (HSYA), the primary bioactive compound isolated from safflower, has also been extensively documented to mitigate disease progression in ischemic stroke and neurodegenerative disorders. Its therapeutic effects are primarily mediated by enhancing angiogenesis [16], suppressing neuroinflammation [17], and alleviating oxidative stress [18,19]. HSYA has been reported to modulate macrophage-induced inflammation by inhibiting the PI3K/AKT/mTOR and NF-κB signaling pathways, thereby reducing plaque formation and inflammatory responses in atherosclerotic mice [20]. In the study by Wei et al., HSYA was shown to enhance the functionality of endothelial progenitor cells (EPCs) through the HO-1/VEGF-A/SDF-1α cascade, thereby promoting myocardial angiogenesis in a mouse model of myocardial infarction (MI) [16].

Hydrogels are widely researched for nerve repair due to their ideal scaffold properties [21,22,23]. Their excellent biocompatibility and degradability support early tissue growth before gradually degrading to aid regeneration [24,25]. Additionally, their 3D network structure mimics the extracellular matrix, allowing the encapsulation of cells, drugs, and growth factors for sustained release [26]. Furthermore, injectable hydrogels enable targeted and controlled drug delivery, overcoming limitations of conventional methods such as poor blood–brain barrier penetration and rapid systemic clearance [27,28]. Among various hydrogel materials, hyaluronic acid (HA) has attracted significant attention due to its outstanding ECM-mimicking properties and crosslinking capability [29]. Broguiere et al. developed a novel enzymatic hydrogel by crosslinking high-molecular-weight HA via the transglutaminase (TG) activity of activated coagulation factor XIII (FXIIIa) [30]. This hydrogel demonstrated robust stability, an absence of cytotoxic effects, and exhibited properties that significantly enhanced neurite outgrowth, as well as facilitated the formation of axons and dendrites in three-dimensional (3D) neuronal culture models. It is noteworthy that incorporating traditional Chinese medicine bioactive compounds, growth factors, or pharmaceutical agents into hydrogels has shown remarkable potential across various tissue regeneration applications. SAA-loaded composite hydrogels have been shown to inhibit the cGAS-STING pathway, thereby reducing inflammation, and reversing disk degeneration [31]. Gao et al. developed a hydrogel for dual delivery of hydroxysafflor yellow A (HSYA) and deferoxamine (DFO) to address impaired healing in diabetic wounds [32]. Their findings demonstrated a marked therapeutic interaction between HSYA and DFO, that is, the combinatorial therapy not only enhanced in vitro angiogenesis (tube formation assay) but also accelerated chronic wound closure in diabetic models, outperforming both single-agent treatments and untreated controls [32]. However, to the best of our knowledge, there have been no studies investigated hydrogel-delivered SAA, HSYA, or their combination for treating brain injury-related disorders.

In this study, we designed an HT hydrogel co-loaded with SAA and HSYA (HT/SAA/HSYA) for TBI repair, leveraging the known anti-inflammatory and pro-angiogenic capacities of SAA and HSYA. The physical properties and bioactivities of the HT and HT/SAA/HSYA hydrogels were systematically evaluated both in vitro and in vivo. Consistent with their known bioactivities, in vitro experiments confirmed significant anti-inflammatory and pro-angiogenic effects of SAA and HSYA. Furthermore, the therapeutic efficacy of HT/SAA/HSYA hydrogels was assessed in a murine TBI model. Collectively, these findings demonstrate that the multifunctional HT/SAA/HSYA hydrogel, endowed with dual anti-inflammatory and pro-angiogenic activities, represents a promising novel therapeutic strategy for TBI treatment.

## 2. Materials and Methods

### 2.1. Materials, Cells, and Animals

Sodium hyaluronate (HA, 200–400 kDa), 2-(N-morpholinyl) ethanesulfonic acid (MES), 1-(3-dimethylaminopropyl)-3-ethylcarbodiimide hydrochloride (EDC), N-hydroxysuccinimide (NHS), and tyramine hydrochloride (TA) were purchased from Aladdin (Shanghai, China). A dialysis bag (MWCO = 3500) was purchased from Biosharp (Hefei, China). Salvianolic acid A (SAA) and hydroxysafflor yellow A were purchased from Aifa Biotechnology (Chengdu, China). Mouse microglia (BV2) and human umbilical vein endothelial cells (HUVEC) were purchased from Procell (Wuhan, China). Fetal bovine serum (FBS) and DMEM basic were supplied by Gibco (Sao Paulo, Brazil).

Male C57BL/6 mice (20–25 g) were provided by Shanghai SLAC Laboratory Animal Co., Ltd. (Shanghai, China). The mice were housed at 25 °C in SPF-level animal facilities, with free access to food and water. All animal experiments were approved by the Zhejiang Chinese Medical University Laboratory Animal Research Center (ZCMULARC).

### 2.2. Synthesis and Characterization of HA-TA

HA-TA was synthesized through a coupling reaction between the carboxyl groups of HA and the amino groups of TA, resulting in the formation of covalent bonds. First, HA (1 g, 1% *w*/*v*) was dissolved in 100 mL of MES buffer. Subsequently, 0.96 g of EDC and 0.575 g of NHS were added, and the mixture was stirred for 1 h to activate the carboxyl groups in HA. Next, 0.87 g of TA was added, and the mixture was stirred at room temperature for 24 h. Finally, the reaction solution was transferred to a dialysis bag (MW = 3500), dialyzed in a 5 g/L NaCl solution for 1 day, followed by dialysis in deionized water for 2 days, and then lyophilized to obtain HA-TA. The product was characterized by ^1^H NMR spectroscopy (600 MHz, Avance III, Bruker, Karlsruhe, Germany) and Fourier-transform infrared spectroscopy (FT-IR) (VERTEX 70v, Bruker, Germany).

### 2.3. Preparation and Physical Characterization of HT Hydrogels

The HT hydrogels were crosslinked through either the C-C bond between the ortho-carbon of the HA-TA aromatic ring or the C-O bond between the ortho-carbon and the phenolic oxygen atom, catalyzed by HRP and H_2_O_2_. Firstly, the HA-TA was dissolved in PBS to prepare solutions at 1%, 2%, and 3% (*w*/*v*). Then, the mixture of HA-TA solution and HRP (3 U/mL), HA-TA solution, and H_2_O_2_ (6 mM) were added into the different chambers of the double-chamber syringe to obtain HT hydrogels at 1%, 2%, and 3%. The corresponding concentrations of SAA and HSYA were added to the HA-TA solution to prepare HT hydrogels loaded with final concentrations of 0.5 mg/mL SAA, 0.5 mg/mL HSYA, and 0.5 mg/mL SAA + 0.5 mg/mL HSYA, which were designated as Blank HT, HT/SAA0.5, HT/HSYA0.5, and HT/SAA/HSYA0.5, respectively.

For morphology characterization, the HT hydrogels were rapidly cooled in liquid nitrogen and freeze-dried, after which cross-sections were obtained. After the cross-section was sputter-coated with gold, the internal microstructure of the HT hydrogels was observed using a field emission scanning electron microscope (SEM, Hitachi SU8010, Tokyo, Japan). Finally, 15 pores were randomly selected from each hydrogel concentration, and their average pore sizes were analyzed using ImageJ 2.14.0 software.

The rheological properties of the hydrogels were evaluated using a rheometer (MCR302, Anton Paar, Graz, Austria) equipped with a PP25 parallel-plate geometry. The precursor solutions of each group were injected between the plates using a dual-chamber syringe, and the tests were immediately performed at 25 °C under a constant strain of 2% and an angular frequency of 10 rad/s. The storage modulus (G′) and loss modulus (G″) of the hydrogels were compared among different groups.

The degradation curve of HT hydrogel in PBS was determined. Hydrogels with known weights were immersed in PBS solution and placed in a constant temperature shaker (37 °C, 50 rpm). The initial weight of the hydrogels was recorded as *W*_0_, and the hydrogels were removed at predetermined time points for measurement. After excess water was removed using filter paper, the hydrogels were weighed, and their weights were recorded as *W_t_*. The measurement was performed in triplicate. The degradation rate (*D*) of the hydrogels was calculated according to Formula (1).

(1) $$D=\frac{W_{0}-W_{t}}{W_{0}}\times 100\%$$

The drug release profiles from the HT hydrogels were investigated using HSYA as a model drug. Briefly, 1 mL of HT/HSYA hydrogels at different concentrations were immersed in 3 mL of PBS solution and incubated in a thermostatic shaker bath at 37 °C and 50 rpm. At predetermined time intervals, 2 mL of supernatant was collected and replaced with an equal volume of fresh PBS solution. All experiments were conducted in triplicate. The supernatant samples were collected, and the concentration of HSYA in the supernatants was measured using an ultraviolet/visible (UV/Vis) spectrophotometer (UNICO-2800, Franksville, WI, USA) at an absorbance wavelength of 403 nm. The release amount of HSYA was recorded as *r*, and the total amount of the loaded HSYA was *l*. The cumulative release rate (*R*) of HSYA was calculated using Formula (2).

(2) $$R=\frac{r}{l}\times 100\%$$

The in vitro drug release profiles were fitted using various mathematical models to evaluate the release kinetics and elucidate the underlying release mechanisms. The applied models included zero-order kinetics, first-order kinetics, the Higuchi model, and the Korsmeyer-Peppas model. The optimal fitting model for describing the drug release behavior was selected based on the highest adjusted coefficient of determination [33].

### 2.4. Evaluation of Cytocompatibility of Hydrogels

To determine the impact of combined SAA and HSYA treatment on cellular proliferation, BV2 cells were seeded in a 96-well plate at 0.5 × 10^4^ cells/well and cultured in a 5% CO_2_ cell incubator at 37 °C for 24 h. Cells were divided into four groups: control, SAA (100 μM), HSYA (50 μM), and SAA (100 μM)/HSYA (50 μM). After 24 h of treatment, the medium was removed, and the cells were washed once with PBS. Afterwards, 100 μL of serum-free DMEM medium containing 10% (*v*/*v*) Cell Counting Kit-8 (CCK-8, Biosharp, Hefei, China) was added to each well, with a blank group (without cells) included. The plates were incubated in the dark at 37 °C for 1 h. The absorbance at 450 nm was measured using a microplate reader (INFINITE 200 PRO, Tecan, Grödig, Austria).

The cytocompatibility of hydrogels with BV2 cells was evaluated using a CCK-8 assay and LIVE/DEAD staining kit (Solarbio, Beijing, China). For the CCK-8 experiments, 1 mL of HT hydrogel was added to 3 mL of DMEM medium and incubated for 24 h to obtain hydrogel extracts. The extracts were then diluted fivefold and filtered through 0.22 μm sterile filters. BV2 cells were seeded in 24-well plates at a density of 1 × 10^4^ cells per well and cultured for 24 h in a 37 °C, 5% CO_2_ incubator. The cells were then treated with hydrogel extracts and further incubated for either 24 or 72 h. Cell viability was quantitatively determined using the CCK-8 assay according to the manufacturer’s protocol.

For the LIVE/DEAD staining assay, BV2 cells were seeded in 24-well plates at a density of 1 × 10^4^ cells per well. Following treatment with the extracts, the cells were cultured for an additional 24 h. All other steps were performed as described above. According to the manufacturer’s instructions, 200 μL of Calcein-AM/PI working solution was added to each well, and the cells were stained in darkness at 37 °C for 20 min. The cells were visualized and imaged under a fluorescence microscope (Leica DMI8, Wetzlar, Germany), with live cells stained green and dead cells stained red.

### 2.5. Matrigel-Based Angiogenesis Assay

Human umbilical vein endothelial cells (HUVEC) were serum-starved overnight in serum-free conditions. Matrigel (Corning, New York, NY, USA) was stored at 4 °C overnight to thaw, while the 96-well plate and pipette tips were stored at 4 °C to pre-cool. Then, 50 μL of the thawed Matrigel was added to each well of the 96-well plate and incubated at 37 °C for 40 min to allow solidification. The cells were assigned to four groups: control, SAA (100 μM), HSYA (50 μM), and SAA (100 μM)/HSYA (50 μM). After resuspending in a complete medium containing drugs, the cells were seeded at 4 × 10^4^ cells/well onto the Matrigel-coated wells. After 6 h of culture, the cells were examined using a microscope and photographed. The number of nodes, meshes, and the total segment length of the tubular structure were calculated using ImageJ 2.14.0 software (Angiogenesis Analyzer plugin).

### 2.6. Inflammatory Effects of SAA and HSYA In Vitro

The inflammatory model was established by stimulating BV2 cells with lipopolysaccharide (LPS) to investigate the regulatory effects of SAA and HSYA on microglia-mediated inflammation. After 24 h of adherent culture, the cells were incubated with 1 μg/mL LPS (Sigma-Aldrich, St. Louis, MO, USA) for 6 h to induce M1 polarization. The drug-administered groups were treated with the corresponding concentrations of SAA (100 μM) or HSYA (50 μM).

For RT-PCR analysis, the cells were divided into 5 groups: control, LPS, LPS + SAA, LPS + HSYA, and LPS + SAA/HSYA. After collecting the processed cells in each group, total RNA was extracted using an RNA extraction kit (Vazyme, Nanjing, China). The total RNA concentration was measured using a NanoDrop ONE microvolume ultraviolet-visible spectrophotometer (Thermo Scientific, Waltham, MA, USA). cDNA was synthesized using a reverse transcription kit (Biosharp, Hefei, China). Finally, RT-qPCR was performed using SYBR^®^ Green Real-Time PCR Master Mix (TOYOBO, Osaka, Japan). The gene expression levels of IL-1β, TNF-α, TLR4, and NF-κB in BV2 cells were analyzed using the 2^−ΔΔCt^ method. The primer sequences are listed in Table 1.

For Western blot analysis, cells were digested with cell lysate (Beyotime, Shanghai, China) on ice for 30 min. The lysed cells were collected and centrifuged at 12,000 rpm for 10 min at 4 °C. The supernatant was collected, and the protein concentration was determined using the BCA kit (Beyotime, Shanghai, China). Proteins were separated by SDS-PAGE and transferred onto PVDF membranes (Millipore, Waltham, MA, USA). The PVDF membrane was blocked with 5% skimmed milk powder (Yamei Bio, Shanghai, China) for 1 h. The membrane was incubated with TLR4 (1:4000, 66350-1-Ig, ProteinTech, Wuhan, China) and NF-κB (1:1000, HY-P80245, MedChemExpress, Monmouth Junction, NJ, USA), gently agitated overnight at 4 °C. The membrane was washed 3 times with TBST solution, 10 min each time. The membrane was incubated with horseradish peroxidase-labeled goat anti-rabbit IgG secondary antibody or goat anti-mouse IgG secondary antibody (ProteinTech, Wuhan, China) at room temperature for 1 h. Protein analysis was visualized and quantified using ImageJ 2.14.0 software.

### 2.7. Cell Immunofluorescence Staining

BV2 cells were seeded onto cell slides, and the other groups were treated as described above. The cells were fixed with 4% paraformaldehyde for 15 min. After washing with PBS 3 times, the cells were incubated with 0.3% Triton-100 for 10 min. The cells were blocked with 5% goat serum for 1 h at room temperature. Then, rabbit clonal antibody CD86 (1:600, GB115630, Servicebio, Wuhan, China) was added and incubated with the cells at 4 °C overnight. Afterwards, Cy3-labeled goat anti-rabbit IgG secondary antibody (1:300, Servicebio, Wuhan, China) was incubated for 1 h. Finally, coverslips were sealed by adding a DAPI-containing anti-fluorescence quencher (Beyotime, Shanghai, China), and the staining effect was observed and photographed under a fluorescence microscope (Leica DMI8, Wetzlar, Germany).

### 2.8. In Vivo Biocompatibility of HT Hydrogels

All animal experiments were conducted by the experimental animal care and use guidelines of Zhejiang Chinese Medical University and were approved by the Animal Ethics Committee of Zhejiang Chinese Medical University.

For histological evaluation, HT hydrogel precursor solution, HRP (3 U/mL), and H_2_O_2_ (6 mM) were filtered through a 0.22 μm sterile filter membrane. SAA (0.5 mg/mL) and/or HSYA (0.5 mg/mL) were loaded to obtain the precursor solutions of Blank HT, HT/SAA0.5, HT/HSYA0.5, and HT/SAA/HSYA0.5. Then, 400 µL of each hydrogel was subcutaneously injected into the back of male Sprague-Dawley (SD) rats using a double-lumen syringe. Before removal, the needle was left in place for 3 min to allow the hydrogel to set. Rats were euthanized on day 14 (D14) and day 28 (D28), and the hydrogels were collected and weighed. The surrounding tissue of the residual hydrogel was separated and fixed in 4% paraformaldehyde. Finally, H&E staining was performed.

Neovascularization in the area around the hydrogel was evaluated by immunofluorescence staining for CD31 and α-SMA. After dewaxing, hydration, and antigen retrieval of paraffin sections, 5% bovine serum albumin (BSA) was added to block nonspecific binding for 30 min. After removing the blocking solution, rabbit clonal antibodies CD31 (1:400 dilution, GB120008, Servicebio, Wuhan, China) and α-SMA (1:600 dilution, GB111364, Servicebio, Wuhan, China) were added and incubated overnight at 4 °C. After washing with PBS, Cy3-labeled goat anti-rabbit IgG secondary antibody (1:300, Servicebio, Wuhan, China), or FITC-labeled goat anti-rat IgG secondary antibody (1:200, GB22302, Servicebio, Wuhan, China) was added and incubated at 37 °C for 1 h. Finally, the slides were mounted with an anti-fade mounting medium containing DAPI. The slides were imaged using a virtual slide microscope (VS120-S6-W, Olympus, Tokyo, Japan) and quantitatively analyzed using ImageJ 2.14.0 software.

### 2.9. Establishment of Mouse TBI Model and Hydrogel Implantation

A TBI model was established using 20–25 g male C57BL/6 mice. After the mice were anesthetized, the hair above the skull was shaved. The mice were secured to the brain stereotaxic device. After exposing the skull and removing the periosteum, a cranial window (3 mm in diameter) was drilled using a skull drill. The meninges were removed, and the cerebral cortex was exposed. The brain parenchyma was aspirated using a flat-head needle (approximately 1 mm in diameter) to create a cylindrical open focal injury with 1 mm in diameter and 1 mm in depth. After bleeding was controlled using sterile cotton swabs and normal saline, HT/SAA, HT/HSYA, and HT/SAA/HSAY hydrogels were injected into the defect area. After the hydrogel had completely solidified, the skull window was covered with sterile bone wax [34]. Finally, the scalp of the mice was sutured, and the postoperative mice were placed on a heating pad to restore body temperature. The mice were euthanized, and their brains were removed on days 0, 14, and 28. The brains were fixed with 4% paraformaldehyde and subjected to H&E staining analysis.

To evaluate the systemic biosafety of the hydrogel, major organs (heart, liver, spleen, lungs, and kidneys) from mice in the HT/SAA/HSYA group were collected on day 28 and subjected to H&E staining, with normal C57 mice serving as the control group.

### 2.10. Statistical Analysis

Statistical analysis was performed using GraphPad Prism 10.1.2. The data are expressed as mean ± standard deviation (mean ± SD). One-way analysis of variance (ANOVA) was used to assess the significance of differences. In all experiments, * *p* < 0.05, ** *p* < 0.01, and *** *p* < 0.001.

## 3. Results

### 3.1. Preparation and Characterization of the Hydrogels

**Figure 1:**
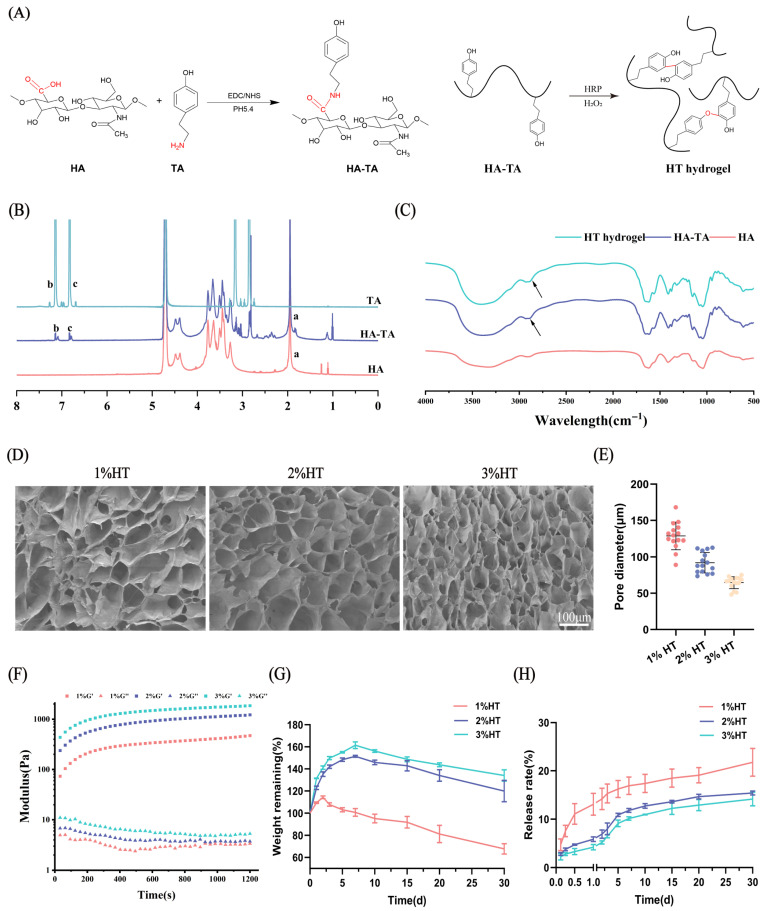
Characterization of the HT Hydrogels. (**A**) Schematic illustration of the synthesis of HA-TA and HT hydrogels. (**B**) ^1^H NMR spectra of HA (red), TA (blue), and HA-TA (purple), a, b and c represent the peaks at chemical shift 1.9, 7.1 and 6.8 ppm, respectively. (**C**) FT-IR spectra of HA, HA-TA, and HT hydrogel. (**D**) SEM images of 1%, 2%, and 3% HT hydrogels (scale bar = 100 µm). (**E**) Pore size distribution of 1%, 2%, and 3% HT hydrogels (*n* = 15). (**F**) The storage modulus (G′) and loss modulus (G″) of 1%, 2%, and 3% HT hydrogels were measured over time at a constant strain of 2% and an angular frequency of 10 rad/s. (**G**) Swelling and degradation behavior of 1%, 2%, and 3% HT hydrogels in PBS. (**H**) Release profile of HSYA from the HT/HSYA hydrogels in PBS. Data are presented as mean ± SD, *n* = 3.

As illustrated in Figure 1A, HA-TA was synthesized through the conjugation of carboxyl groups in hyaluronic acid (HA) with amino groups in tyramine (TA), mediated by EDC/NHS. The HT hydrogel is formed through crosslinking via either C-C bonds between aromatic carbocations or C-O bonds between carbocations and phenolic oxygen atoms in the HA-TA system [35]. The successful synthesis of HA-TA was confirmed by ^1^H NMR in Figure 1B. A characteristic peak of HA was observed at position ‘a’. Compared with HA, the spectra of HA-TA exhibited new peaks (b and c) at chemical shifts (δ) 7.1 and 6.8 ppm, corresponding to the characteristic proton peaks of the phenol groups in TA, confirming that TA was successfully grafted to the chain of HA.

The FT-IR spectra of HA, HA-TA, and the HA-TA (HT) hydrogel are presented in Figure 1C. A distinct absorption peak at 2930 cm^−1^ is observed for both HA-TA and the HT hydrogel, which can be ascribed to the aromatic C–H stretching vibration resulting from the incorporation of TA groups in HA-TA. However, the infrared spectra of the HT hydrogel and HA-TA are fundamentally similar. This phenomenon can be attributed to the fact that HT hydrogel formation occurs through covalent crosslinking via either C–C bonds between adjacent aromatic carbons or C–O bonds between phenolic oxygens in HA-TA, without generating new chemical bonds. Similar findings have been reported in previous studies [36].

The microstructures of the hydrogels at different concentrations are depicted in Figure 1D. Firstly, all hydrogels exhibited a porous and interconnected microscopic structure. In addition, the pore size of the hydrogels decreased with increasing hydrogel concentration. Figure 1E quantitatively summarizes the pore size of HT hydrogels with different concentrations. The quantified pore size of 1% HT, 2% HT, and 3% HT hydrogels were 128.77 ± 18.81 μm, 92.01 ± 14.16 μm, and 64.51 ± 8.21 μm. Figure 1E reflects the negative correlation of precursor concentration on gelation time of hydrogel formation with gelation time of 87.33 ± 2.08 s, 37.67 ± 1.15 s, and 16 ± 1.73 s for 1% HT, 2% HT, and 3% HT hydrogels, respectively.

The rheological properties of the hydrogels were investigated using a rheometer. As shown in Figure 1F, under fixed frequency (10 rad/s) and strain (2%) conditions, the storage modulus (G′) of the hydrogels increased with concentration from 1% to 3%. All samples reached a plateau in G′ values after approximately 1000 s, indicating complete formation and stabilization of the hydrogel network structure. Finally, the hydrogels at 1%, 2%, and 3% achieved storage modulus (G′) of 472 Pa, 1220 Pa, and 1860 Pa, respectively.

The degradation behavior of the HT hydrogel was measured by immersing the hydrogels in PBS solution and placing them in a thermostatic shaker at 37 °C, 50 rpm. All hydrogels exhibited similar swelling degradation characteristics as shown in Figure 1G, with 1% HT degrading the fastest, followed by 2% HT and 3% HT. During the 30 days of the experiment, none of the hydrogel were completely degraded.

The release rates of HSYA from 1%, 2%, and 3% HT hydrogels were measured under the same conditions. As depicted in Figure 1H, all hydrogels released HSYA steadily and continuously over time, without any obvious burst release behavior. In addition, the release rate of HSYA from hydrogels decreased with increasing the hydrogel concentration.

To gain deeper insight into the release mechanisms, the HSYA release profiles from various hydrogels were analyzed using four kinetic models: zero-order kinetics, first-order kinetics, Higuchi model, and Korsmeyer-Peppas model [33]. The model with the highest correlation coefficient (R^2^) was considered the most appropriate for describing the release behavior. As presented in Table 2, the Korsmeyer-Peppas model exhibited the optimal fitting performance for all hydrogel formulations, with adjusted R^2^ values of 0.926, 0.983, and 0.987 for 1% HT, 2% HT, and 3% HT hydrogels, respectively. The ranking of model suitability was established as: Korsmeyer-Peppas model > Higuchi model > First-order model > Zero-order model.

### 3.2. Cytocompatibility Evaluation of the Hydrogels

**Figure 2:**
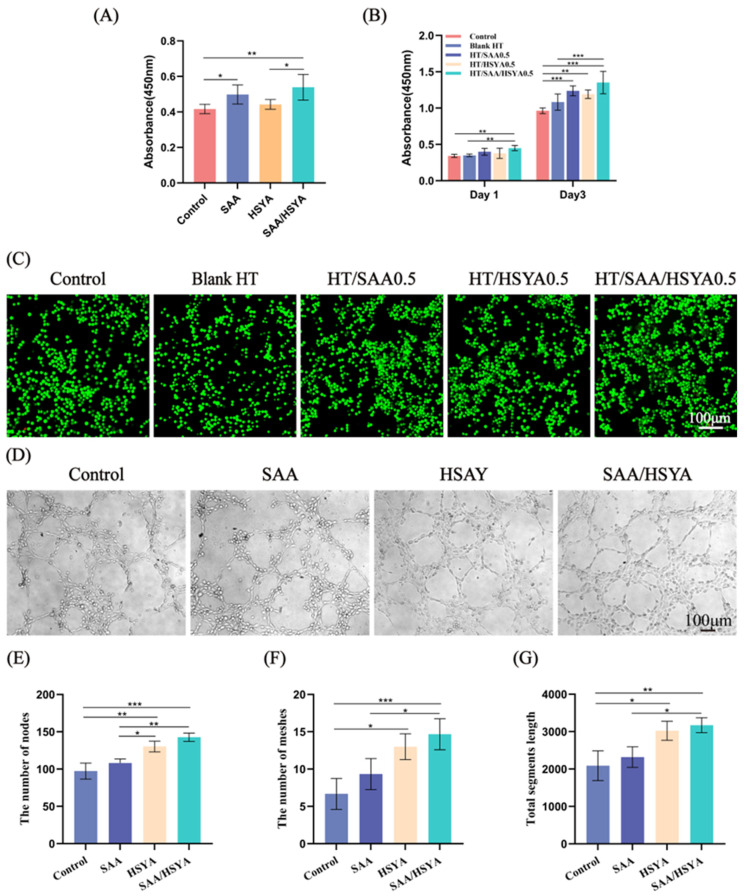
(**A**) Effect of SAA and HSYA combination on BV2 cell proliferation. (**B**) The CCK-8 assay evaluated the cytotoxicity of blank HT hydrogel and HT hydrogels loaded with SAA and/or HSYA toward BV2 cells. (**C**) Live/dead staining evaluated the biocompatibility of blank hydrogels and various drug-loaded hydrogels with BV2 cells, where green fluorescence indicated viable cells (scale bar = 100 µm). (**D**) Effect of SAA and HSYA on HUVEC angiogenesis (scale bar = 100 µm). (**E**) Number of nodes, (**F**) number of meshes, and (**G**) quantification of total segment length, representing the ability of each group of cells to form lumens in vitro. Data are represented as mean ± SD, *n* = 3, * *p* < 0.05, ** *p* < 0.01, *** *p* < 0.001.

The effects of the SAA and HSYA combination on cell proliferation and the cytocompatibility of the hydrogels were evaluated using CCK-8 and LIVE/DEAD staining assays. The CCK-8 results (Figure 2A) indicate that none of the administered groups exhibited cytotoxicity toward BV2 cells. Compared to the control group, the HSYA group showed no significant difference in cell viability. Both the SAA group and the SAA/HSYA group demonstrated varying degrees of promotion in cell proliferation. Furthermore, the SAA/HSYA group exhibited a more significant enhancement in cell proliferation capability relative to the groups treated with SAA or HSYA alone. Figure 2B,C demonstrate the biocompatibility evaluation of blank HT hydrogel and HT hydrogels loaded with SAA and/or HSYA. CCK-8 assay results (Figure 2B) revealed that after 1-day culture, no significant differences in cell viability were observed among Blank HT, HT/SAA0.5, and HT/HSYA0.5 groups compared to the control. However, the HT/SAA/HSYA0.5 group showed significantly higher absorbance values than the control group. After 3 days of culture, BV2 cells cultured in hydrogel extracts demonstrated normal proliferation. The blank HT group maintained cell viability comparable to the normal control group, while all drug-loaded hydrogels exhibited varying degrees of proliferative enhancement, with the HT/SAA/HSYA0.5 group showing the most pronounced effect. These results indicate that all HT hydrogels possess excellent long-term cytocompatibility, and the HT/SAA/HSYA hydrogels can effectively promote BV2 cell proliferation. In addition, the LIVE/DEAD staining results in Figure 2C displayed prominent green fluorescence (viable cells), indicating that all hydrogel groups exhibited excellent cell compatibility. Notably, the HT/SAA/HSYA0.5 group demonstrated higher green fluorescence intensity compared to the control group, further substantiating the promotive effect of the HT/SAA/HSYA hydrogel on the proliferation of BV2 cells. These findings are consistent with the results obtained from the CCK-8 assay.

### 3.3. In Vitro Angiogenesis Assay

The Matrigel assay is a classic and widely utilized experimental methodology that enables rapid and quantitative evaluation of in vitro angiogenesis [37]. Therefore, the Matrigel experiment was conducted to verify the effects of SAA, HSYA, and their combination with HUVEC tube formation. As shown in Figure 2D, there was no significant difference in the SAA group compared to the normal group. However, the number of nodes, the number of meshes, and the total segment length (Figure 2E–G) were significantly increased in the HSYA and SAA/HSYA groups. The results demonstrate that both HSYA and the SAA/HSYA combination exhibit a significant capacity to promote lumen formation.

### 3.4. In Vitro Inflammatory Regulation

**Figure 3:**
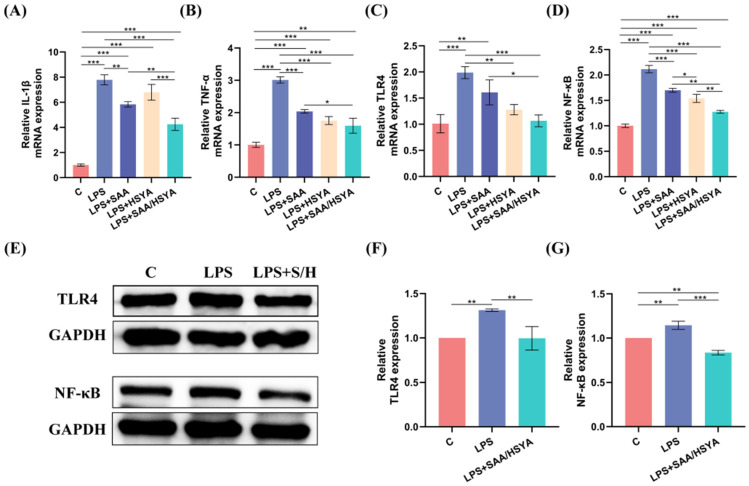
The mRNA expression levels of (**A**) IL-1β, (**B**) TNF-α, (**C**) TLR4, and (**D**) NF-κB in SAA, HSYA, and co-administration-treated BV2 cells after LPS stimulation. (**E**) Protein expression levels of TLR4 and NF-κB. Quantitative analysis of Western blot results of (**F**) TLR4, and (**G**) NF-κB. Data are represented as mean ± SD, *n* = 3; * *p* < 0.05, ** *p* < 0.01, *** *p* < 0.001.

To simulate the inflammatory conditions in TBI, an LPS-induced BV2 inflammation model was established. RT-PCR, Western blotting, and immunofluorescence staining experiments were performed to investigate and analyze whether SAA and HSYA could exert anti-inflammatory effects by regulating the TLR4/NF-κB signaling pathway. After LPS modeling, the mRNA expression levels of the pro-inflammatory cytokines TNF-α and IL-1β were significantly increased in the LPS group compared to the control group. By contrast, the treatment with SAA, HSYA, and SAA/HSYA significantly reduced the overexpression of TNF-α and IL-1β. Moreover, the expression levels of inflammatory factors were further reduced in the SAA and HSYA combination group compared to the groups administered alone, indicating the superior efficacy of combination of SAA and HSYA (Figure 3A,B).

To elucidate the anti-inflammatory mechanisms of SAA and HSYA, TLR4, and NF-κB expression levels were examined by RT-PCR and Western blot analysis. As shown in Figure 3C,D, compared with the normal control group, the LPS-treated group exhibited significantly elevated mRNA expression levels of both TLR4 and NF-κB. Following drug interventions, all treatment groups showed varying degrees of reduction in TLR4 and NF-κB mRNA expression. Notably, the SAA/HSYA combination group demonstrated more pronounced inhibitory effects compared to single-agent treatment groups. These results suggest that SAA and HSYA may cooperatively suppress the inflammatory cascade through modulation of the TLR4/NF-κB signaling pathway. As shown in Figure 3E–G, the protein expression levels of TLR4 and NF-κB were significantly higher in the LPS modeling group compared to the control group. Both TLR4 and NF-κB expression were suppressed after SAA/HSYA administration compared to the LPS group. These results indicate that SAA and HSYA might exert their anti-inflammatory effects by inhibiting the TLR4/NF-κB signaling pathway.

**Figure 4:**
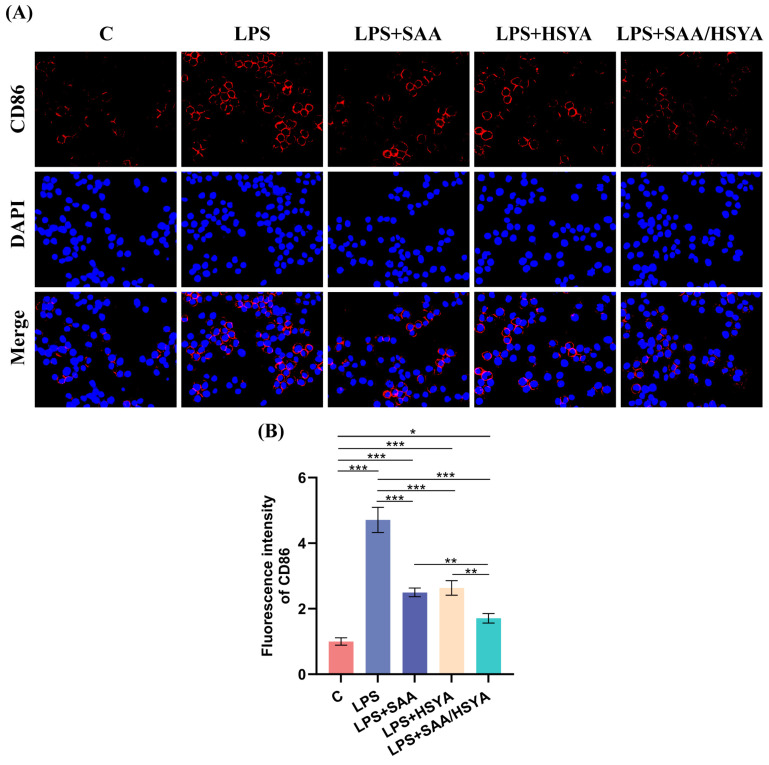
Effects of SAA, HSYA, and SAA/HSYA on LPS-stimulated microglia polarization. (**A**) Immunofluorescence staining of CD86-positive cells (red) and DAPI (blue) in microglia. (**B**) Quantification of the relative fluorescence intensity of CD86 (scale bar = 50 µm). Data are represented as mean ± SD, *n* = 3; * *p* < 0.05, ** *p* < 0.01, *** *p* < 0.001.

Microglial polarization plays a pivotal role in neuroinflammatory responses. Under pathological conditions, microglia are activated and polarized into the pro-inflammatory M1 phenotype, accompanied by a significant upregulation of CD86 expression. In this study, microglia were immunofluorescence-stained with CD86 (red) and DAPI (blue). Compared to the control group, the immunofluorescence intensity of CD86 was significantly elevated in the LPS group. Conversely, all treatment groups demonstrated a reduction in immunofluorescence intensity relative to the LPS group, with the most substantial decrease observed in the SAA/HSYA group (Figure 4B). These findings suggest that SAA and HSYA administration can inhibit microglia M1 polarization and prevent the conversion of microglia into a pro-inflammatory phenotype. In conclusion, the RT-PCR, Western blotting, and immunofluorescence staining results indicated that the administration of SAA and HSYA could exert an inhibitory effect on inflammation by modulating the TLR4/NF-κB signaling pathway, with the therapeutic effect of the combination being more pronounced than that of individual administrations.

### 3.5. Evaluation of In Vivo Compatibility of Hydrogels

**Figure 5:**
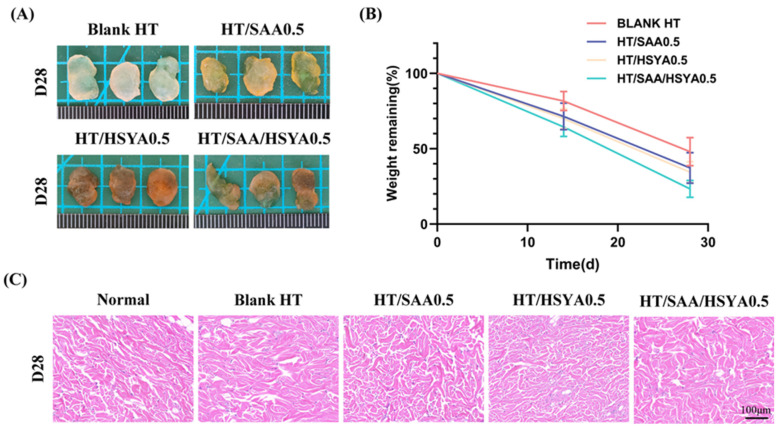
In situ subcutaneous implantation of hydrogels to assess the biocompatibility of blank HT and drug-loaded hydrogels. (**A**) The hydrogels of each group were removed from the subcutaneous tissue of mice on day 28. (**B**) Statistical results of the remaining weight of hydrogels on days 14 and 28 after subcutaneous injection. (**C**) H&E staining (scale bar = 100 μm) of the skin tissue surrounding the subcutaneous injection site of the hydrogels. Data are represented as mean ± SD, *n* = 3.

The biocompatibility was evaluated by subcutaneous injection of 400 μL Blank HT, HT/SAA0.5, HT/HSYA0.5, and HT/SAA/HSYA0.5 hydrogels into the dorsal region of rats. After subcutaneous injection of the hydrogels, the experimental rats were fed, moved normally, and exhibited no bleeding or swelling. The rats were euthanized on days 0, 14, and 28, and the subcutaneous hydrogels were removed and weighed. The degradation characteristics of HT hydrogels in rats were assessed over 28 days. The hydrogels in each group were not completely degraded. The degradation rate of Blank HT was the lowest, whereas the degradation rate of HT/SAA/HSYA0.5 was the highest (Figure 5A,B).

To further analyze the in vivo compatibility of the hydrogel, skin tissue surrounding the hydrogel was collected on day 14 for H&E staining. The results showed that there was no evident inflammatory response in the skin tissue surrounding each hydrogel (Figure 5C), suggesting that the HT hydrogel exhibits good in vivo compatibility and holds great potential for further in vivo applications.

**Figure 6:**
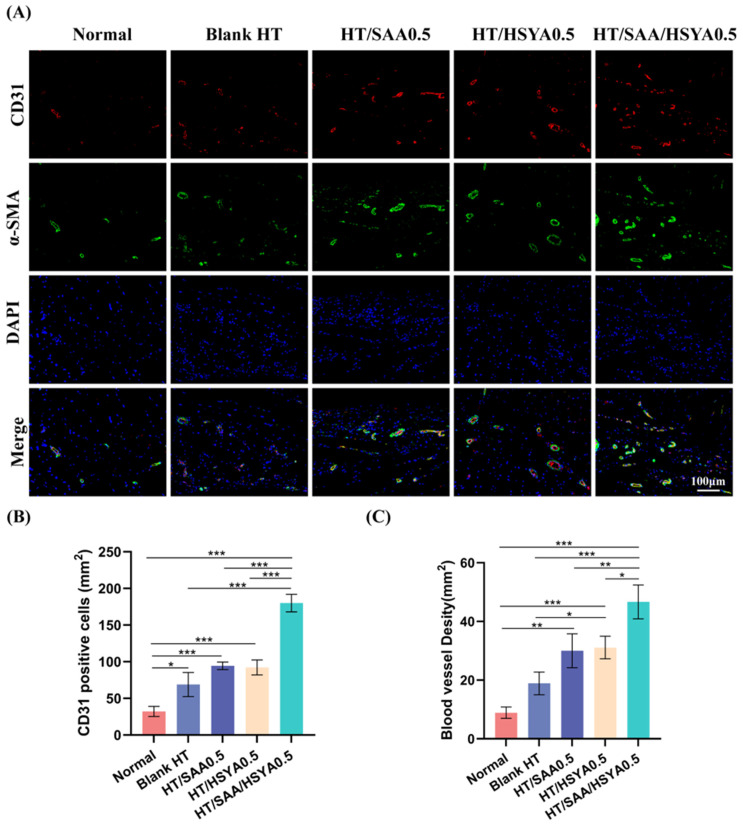
Immunofluorescence staining was used to detect the pro-angiogenic effects of the hydrogels in each group. (**A**) Immunofluorescence staining images showing CD31 (red), α-SMA (green) and DAPI (blue) in the skin tissues surrounding the hydrogels of each group on day 14 after subcutaneous injection (scale bar = 100 µm). (**B**) Quantitative plots showing the number of CD31-positive cells in the perigee skin tissue and (**C**) blood vessel density. Data are represented as mean ± SD, *n* = 3; * *p* < 0.05, ** *p* < 0.01, *** *p* < 0.001.

The pro-angiogenic ability of the hydrogel was evaluated using immunofluorescence staining. As shown in Figure 6A, neovascularization was observed in the skin tissues surrounding Blank HT, HT/SAA0.5, HT/HSYA0.5, and HT/SAA/HSYA0.5. This was characterized by the visualization of CD31-positive cells (red) surrounded by α-SMA-positive cells (green). The mean intensity of CD31-positive cells and quantitative data on vascular density are shown in Figure 6B,C. Compared to the Blank HT group, the expression levels of CD31 and α-SMA in the skin tissues surrounding the hydrogel were significantly elevated in the HT/SAA, HT/HSYA, and HT/SAA/HSYA0.5 groups, with the most notable enhancement observed in the HT/SAA/HSYA0.5 group. These results indicate that the HT/SAA/HSYA0.5 hydrogel possesses remarkable pro-angiogenic capabilities.

### 3.6. HT/SAA/HSYA0.5 Hydrogel Promotes Repair of Traumatic Brain Injury in Mice

**Figure 7:**
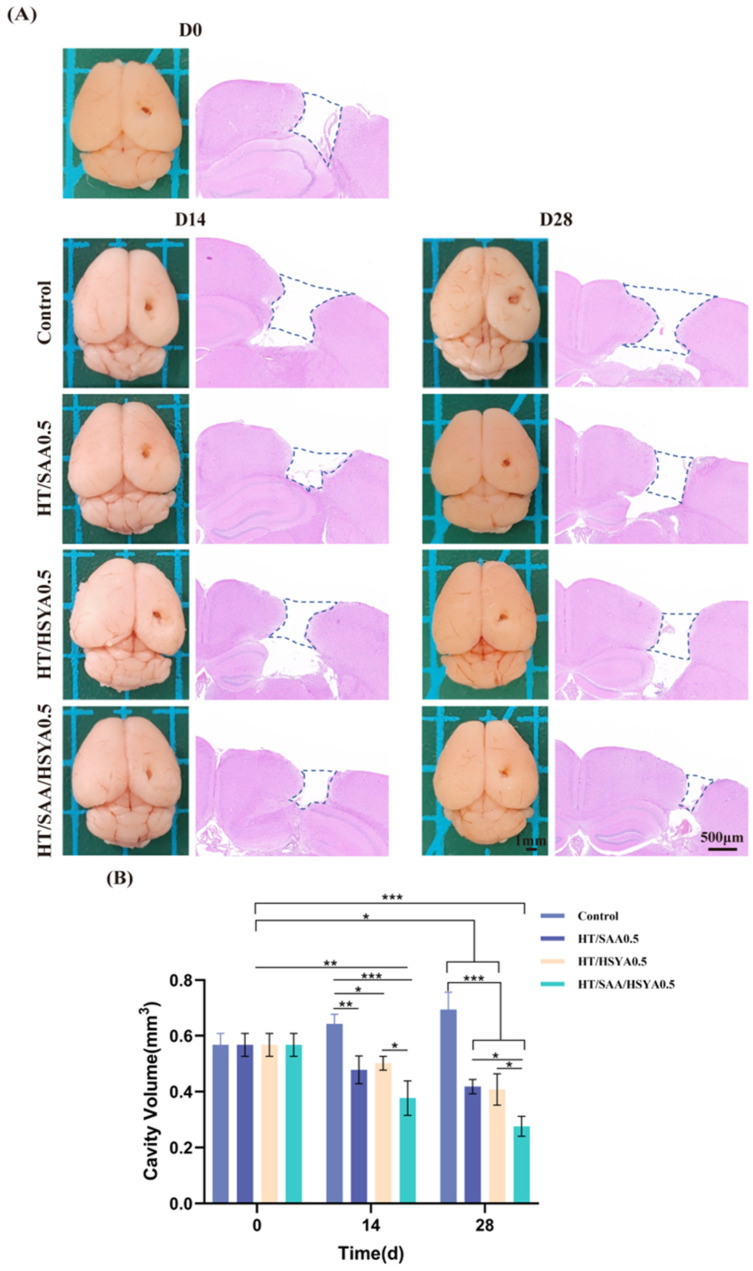
(**A**) The macroscopic observations (scale bar = 1 mm) and H&E staining of brain sections (scale bar = 500 μm) were performed on mouse brains on days 0, 14, and 28. (**B**) Quantitative analysis of the changes in the damaged volume of a mouse brain on days 0, 14, and 28 after hydrogel injection. Data are represented as mean ± SD, *n* = 3; * *p* < 0.05, ** *p* < 0.01, *** *p* < 0.001.

After subcutaneous injection on days 0, 14, and 28, the mice were euthanized, and their brains were collected. Macroscopic examination revealed that, compared to the control group, there was a reduction in the defect area in each drug-loaded hydrogel group on days 14 and 28 (Figure 7A). The volume of damaged tissue in each group was then evaluated using H&E staining. The largest defect area in the coronal section was uniformly selected for statistical analysis, and the defect area extending from the cortex to the hippocampus was measured. The volume of the defect area was then calculated by multiplying the diameter measured on the defect surface. The quantitative data are shown in Figure 7B. Compared with the control group, the defect volume of each drug-loaded hydrogel group was reduced to varying extents on days 14 and 28. The HT/SAA/HSYA0.5 hydrogel group exhibited the most pronounced efficiency and resulted in the smallest damaged volume. These results indicate that the implantation of HT/SAA/HSYA0.5 hydrogel can promote the repair of traumatic brain injury.

**Figure 8:**
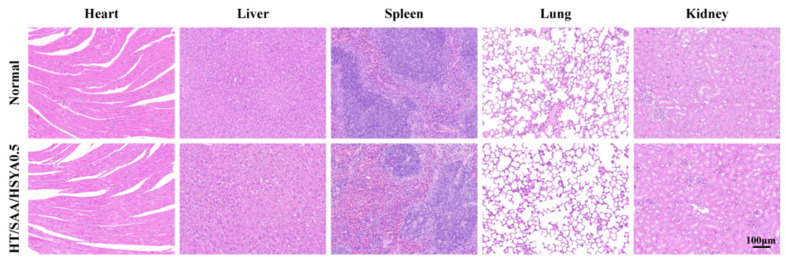
Systemic biocompatibility assessment of each hydrogel group in mice. H&E staining of mouse heart, liver, spleen, lungs, and kidneys after 28 days of treatment.

Finally, the systemic biocompatibility of HT/SAA/HSYA0.5 hydrogel in vital organs such as the heart, liver, spleen, lungs, and kidneys was further evaluated using H&E staining. No obvious tissue damage or morphological changes were observed in the major organs of the experimental group of mice (Figure 8), indicating that the HT hydrogel exhibited no obvious biotoxicity.

## 4. Discussion

Traumatic brain injury (TBI) represents a severe neurological trauma that directly causes structural and functional impairments of brain tissue, resulting in high rates of disability and mortality among patients. Current clinical treatments primarily rely on surgical interventions combined with neurorehabilitation training, yet there remains a lack of effective treatment targeting either primary or secondary injuries in TBI [38]. In recent years, hydrogel-based delivery systems loaded with various bioactive substances have emerged as a promising therapeutic strategy for TBI management.

In our study, we synthesized and optimized an injectable hyaluronic acid-based hydrogel (HT hydrogel) through horseradish peroxidase (HRP)-mediated crosslinking, which serves as a neural scaffold for the co-delivery of SAA and HSYA in TBI treatment. An ideal hydrogel scaffold should meet the following criteria: interconnected porous architecture, appropriate mechanical properties, degradability, and excellent biocompatibility [39]. SEM analysis revealed that the HT hydrogels with varying concentrations exhibited a porous and interconnected microstructure. This architectural characteristic facilitates cell migration, nutrient transport, and metabolic waste removal, thereby creating a favorable microenvironment to support the survival, proliferation, and neurite extension of cerebral cells at the injury site [40]. The matching of hydrogel mechanical properties with those of native tissues can effectively reduce frictional irritation to surrounding tissues after implantation. Previous studies have reported that the elastic modulus of brain tissue typically ranges from 1 to 3 kPa [41,42,43]. The prepared 1%, 2%, and 3% HT hydrogels exhibited storage moduli (G′) of 472 Pa, 1220 Pa, and 1860 Pa, respectively, matching well the mechanical characteristics of natural neural tissues. The degradation profile of hydrogels is critical as it creates space and releases encapsulated drugs during decomposition, thereby facilitating tissue regeneration [44]. Specifically, the HT/SAA/HSYA hydrogel exhibited favorable in vivo degradation kinetics, retaining 23.3% of its initial mass after 28 days. This degradation profile is functionally comparable to that reported by Zhu et al., where an HA-SH/Gel-SH crosslinked hydrogel showed incomplete degradation after 42 days in the brain striatum, as monitored by CEST MRI and dual-color NIR imaging [45]. Immediately post-injection, the hydrogel provides essential structural support to the traumatic brain injury (TBI) site. This support is then complemented by a gradual degradation process that evolves over space and time. Together, this spatiotemporal degradation dynamic fosters a continuously adaptable 3D microenvironment, which is highly conducive to tissue regeneration. Furthermore, live/dead staining and hematoxylin-eosin (HE) staining of major organs (heart, liver, spleen, lung, and kidney) in mice after subcutaneous hydrogel injection confirmed the excellent biocompatibility of the HT hydrogel. Collectively, these results demonstrate the superior performance of our HT hydrogel as a scaffold carrier for neural tissue repair.

Following TBI, the hyperactivated neuroinflammatory response serves as a key pathological mechanism exacerbating secondary brain damage. As the primary immune effector cells in the central nervous system (CNS), microglia are rapidly activated and polarized toward the pro-inflammatory M1 phenotype during the early phase of TBI [46,47]. In our study, LPS stimulation of BV2 microglia was used to simulate the inflammatory environment in the brain. Experimental results demonstrated that in the LPS-induced BV2 microglial inflammation model, SAA and HSYA modulated the TLR4/NF-κB signaling pathway, significantly suppressing the expression of pro-inflammatory cytokines IL-1β and TNF-α while reducing the levels of the M1 microglial marker CD86. Overall, the results suggest that SAA and HSYA may alleviate post-TBI neuroinflammation and promote brain tissue and functional recovery by modulating the TLR4/NF-κB pathway.

Angiogenesis represents a critical mechanism in TBI repair. TBI-induced vascular system damage leads to microcirculatory dysfunction and subsequent tissue ischemia-hypoxia [48]. Prolonged ischemic conditions not only trigger neuronal degeneration and necrosis but also disrupt the formation of a neuroregenerative microenvironment [49]. Consequently, promoting the reconstruction of microvascular networks within the injured region holds significant therapeutic value for both neuronal survival and functional recovery. In our study, the HT/SAA/HSYA hydrogel demonstrated remarkable pro-angiogenic capabilities, as evidenced by significant improvements in tube formation parameters (including node number, mesh number, and trunk length) in the Matrigel assay, along with upregulated expression of vascular markers CD31 and α-SMA in the surrounding skin tissue following subcutaneous injection in rats. These findings provide crucial experimental evidence for developing vascular repair-based therapeutic strategies for TBI.

Finally, the hydrogel was injected into the brain defect site of TBI mice. The H&E staining results demonstrated that, after 28 days post in situ injection, the lesion volume in each hydrogel-treated group was reduced to varying extents, with the HT/SAA/HSYA group exhibiting the most substantial reduction. The observed therapeutic effects might arise from several aspects. Firstly, the in situ injection of hydrogel can provide spatial support for the defect cavity during the early stage of TBI [50]. In addition, SAA and HSYA released during the degradation of the hydrogel can effectively mitigate local inflammatory responses and enhance angiogenesis, thereby ameliorating the hostile microenvironment of the injured area. This dual mechanism not only accelerates the reduction in injury volume but also establishes a conducive environment for potential neurological recovery. In summary, the HT/SAA/HSYA hydrogel demonstrates multifaceted therapeutic potential by providing physical support, inhibiting neuroinflammation, and promoting angiogenesis.

## 5. Conclusions

In this study, we addressed the critical unmet need for effective therapeutic strategies after TBI by developing a novel multifunctional hydrogel, HT/SAA/HSYA. The HT/SAA/HSYA hydrogel demonstrated optimal physicochemical characteristics, including a porous structure, sustained drug release kinetics, excellent biocompatibility in vitro and in vivo, and desirable biodegradability. Our findings robustly demonstrate the effective therapeutic effects of the HT/SAA/HSYA hydrogel through a dual mechanism of suppressing neuroinflammation and promoting angiogenesis. The TBI mouse model confirmed that HT/SAA/HSYA effectively reduced the lesion volume following TBI. Collectively, these results establish the HT/SAA/HSYA composite hydrogel as a highly promising option for TBI treatment. While promising, these findings represent a preliminary investigation. Our study has several limitations: the therapeutic efficacy of the HT/SAA/HSYA hydrogel in promoting functional neurological recovery after TBI and the complete molecular mechanisms underpinning its effects require further elucidation. Future studies will prioritize evaluating neurofunctional outcomes using comprehensive neurobehavioral tests. Furthermore, detailed investigation into the hydrogel’s specific impact on neuroinflammation pathways (beyond established TLR4/NF-κB inhibition) and vascular barrier integrity is essential to fully clarify the molecular mechanisms driving enhanced brain injury repair.

## Figures and Tables

**Table 1:** The sequences of cell RT-qPCR primers.

Primer Name	Primer Sequence (5′-3′)
Mouse IL-1β	F: TGGTGTGTGACGTTCCCATT R: TGTCGTTGCTTGGTTCTCCT
Mouse TNF-α	F: TGTCGTTGCTTGGTTCTCCT R: GGCCAGTGAGTGAAAGGGACA
Mouse TLR4	F: TGGGTCAAGGAACAGAAGCA R: ATCCAACACTAAGGAGGTATTCATC
Mouse NF-κB	F: TGGACGACTCTTGGGAGAAGG R: AACACAGGCTCATACGGTTTCC
Mouse GAPDH	F: TGTGTCCGTCGTGGATCTGA R: TTGCTGTTGAAGTCGCAGGAG

**Table 2:** Correlation coefficients (R^2^) of different kinetic models for hydrogel drug release.

Hydrogel	Zero Order Model	First Order Model	Higuchi Model	Korsmeyer-Peppas Model
	R^2^	R^2^	R^2^	R^2^
1%HT	0.600	0.919	0.947	0.926
2%HT	0.787	0.919	0.816	0.983
3%HT	0.892	0.945	0.984	0.987

## Data Availability

The data that support the findings of this study are available on request from the corresponding author.

## Abbreviations

The following abbreviations are used in this manuscript:

TBI	Traumatic brain injury
HA	Hyaluronic acid
SAA	Salvianolic acid A
HSYA	Hydroxysafflor yellow A
HRP	Horseradish peroxidase
TCM	Traditional Chinese Medicine
MMP-9	Matrix metalloproteinase-9
EPCs	Endothelial progenitor cells
VEGF	Vascular endothelial growth factor
DFO	Deferoxamine
BV2	Mouse microglia
HUVEC	Human umbilical vein endothelial cells
FBS	Fetal bovine serum
SEM	Scanning electron microscope
LPS	Lipopolysaccharide
H&E	Hematoxylin and eosin
